# Direct visualization of the reaction transformation and signal amplification in a DNA molecular machine with total internal reflection fluorescence microscopy

**DOI:** 10.3389/fchem.2013.00023

**Published:** 2013-10-31

**Authors:** Rui Ren, Haiyan Wang, Rui Liu, Shusheng Zhang

**Affiliations:** ^1^School of Chemistry and Chemical Engineering, Linyi University, Linyi, China; ^2^College of Chemistry and Molecular Engineering, Qingdao University of Science and Technology, Qingdao, China

**Keywords:** total internal reflection fluorescence microscopy, direct visualization, DNA molecular machine, rolling circle amplification, cycling amplification reaction

## Abstract

In this study, as a proof of concept, the signal amplification in an artificial DNA molecular machine was directly visualized via total internal reflection fluorescence microscopy (TIRFM). The molecular machine brought about obvious morphology change in DNA nanostructures as well as signal amplifications. On one hand, through a triggered and autonomically repeated RCA, a DNA nano-complex featuring a “locked” circular DNA template (serving as raw feed) was converted into a long periodically repeated strand, i.e., the RCA products. On the other hand, this RCA was repeated in three controllable reaction phases, bring about progressive signal amplification. It was testified that the RCA products (presented as long thread-like fluorescent objects) can be easily distinguished from the inputted DNA probes (presented as fluorescent dots), thus the transformation in reaction can be visualized. Also, by quantitive counting of the aforementioned fluorescence objects, the progress of the reaction through the phases, along with time, and over the lysozyme concentration can be demonstrated through TIRFM visualization. Overall, it was demonstrated that TIRFM is an efficient approach to quantitatively visualize the biochemical processes at single-molecule level.

## Introduction

In recent years, artificial DNA molecular machines have attracted extensive attention for they provided effective signal amplification, which is always highly desirable in bioanalysis. A DNA molecular machine is a single-molecule-scaled, rationally developed system of DNA structural components and DNA reactions that behaves just like a machine: it usually consists of *“parts”* (structural components) made up of DNA strands, carries out “*movements*” based on DNA-specific reactions, responds to “*inputs*” from external stimuli that interact with DNA, brings about “*outputs*” through producing new DNA or transforming existed DNA nanostructures; and quite like macro-scaled machines, DNA molecular machines usually fulfill specific functions based on certain mechanisms. When the stimuli are inputted nucleic acid strands or other biological molecules (to be detected), and when the output was multiplied DNA segments that served as signal, the molecular machine fulfilled the signal amplification function. For example, in *rolling circle amplification (RCA)* (Lizardi et al., 1998; Cheglakov et al., 2007), a DNA machine traditionally adopted for signal amplification, the starting structure was a closed circular template and a primer, and the product was a long strand product with periodically repeated units. As for *nicking-polymerization cycle (NPC)* (Weizmann et al., 2006; Beissenhirtz et al., 2007; Shlyahovsky et al., 2007) developed by Itamar Willner's group, a double-stranded DNA with a nicking site served as the starting structure; in the presence of polymerase and nicking endonuclease that is specific to the nicking site, ssDNA strands can be repetitively produced and released. There was also *target-displacement polymerization (TDP)*, in which the starting structures included complex of a non-nucleic acid target and its aptamer strand and a primer that binds to the former; and in the polymerization that use the aptamer strand as the template, the target was replaced and released, so that it could be recycled (Ren et al., 2012); Also, two or more DNA molecular machines can be interconnected: the product or the intermediate of one machine can act as the “trigger” of another, resulting in dual signal amplification: the downstream cycle poses a further amplification upon the amplification in the upstream cycle (Connolly and Trau, 2010; Ren et al., 2012; Zhao et al., 2013).

TIRF (total internal reflection fluorescence) is a typical direct fluorescence visualization technique at single molecule level; TIRF is different from the traditional fluorescence imaging approaches in its high sensitivity and high signal-noise ratio, which would be necessary for single-molecular imaging. In TIRF, only fluorescent objects in a thin layer (usually less than 200 nm) adjacent to the imaging surface could be excited by the incident laser, and thus are visualizable; while all the other substances (mostly the bulk solution) would not be excited and thus invisible; in this way, the background noise could be suppressed to the most; and, coupled with ultra-high sensitive EMCCD (Electron Multiplying Charge Coupled Device) as the detector of the fluorescence, the signal/noise ratio could be improved to a significant degree. Through TIRF, visualization of fluorescently labeled single molecule would be possible, for example, DNA polymerization in ZMW (Eid et al., 2009), protein translation in ZMW (Uemura et al., 2010), the conformation change of protein due to its interaction with ATP (Zhou et al., 2012) and single-molecular pull-down (Jain et al., 2011).

Until now, the signal amplification in the DNA molecular machines were usually investigated as a whole; while approaches that interrogate the reaction process at single molecule level would reveal more details about molecular machines. On the other hand, although plenty of studies concerning the single-molecular direct visualization have been reported, few of them was concerning the process of an artificial DNA molecular machine, which have been studied for nearly 10 years (Diez et al., 2003). In this study it is proposed to use TIRF to investigate the amplification in a DNA molecular machine.

## Results and discussions

### Design of the molecular machine: main task

The DNA molecular machine was designed to fulfill two functions: when been activated by lysozyme (input), (1) RCA was triggered to produce long strand products (output), (2) the RCA was repeated for numerous times to produce multiplied RCA products, i.e., long DNA strands with periodic repeated sequences. The former was designed to provide a morphology change that can be visualized with TIRF and the later provided the amplification that was studied. Taking advantage of our previous studies on DNA molecular machines (Ren et al., 2012), the two functions were integrated in one-pot mode DNA molecular machine.

### Design of the structural components (“parts” of the molecular machine)

As the core structural component (“part”) of the molecular machine, one single DNA probe was established to support the aforementioned two functions, and two primers, RCA primer and TDP primer, were also used in the molecular machine (to start polymerizations). For the RCA function, the probe featured a “blocked” circular DNA template—it was “blocked” by an aptamer strand, so that it cannot act as a template for RCA reaction at the initial state; and for the multi-phase amplification function, the two strands annealed to each other in two separated segments: a binding site for the RCA primer on the circle template, and a binding site for the TDP primer on the triggering strand, which were both blocked (see section S2 and scheme S1 in Supplementary Materials for details); a potential nicking site of nicking endonuclease (NEase) was designed on the aptamer strand, which would form an actual nicking site when a DNA duplex was formed in the polymerization using the aptamer strand as the template.

### The reactions—the “movements” of the machine

**Scheme 1:**
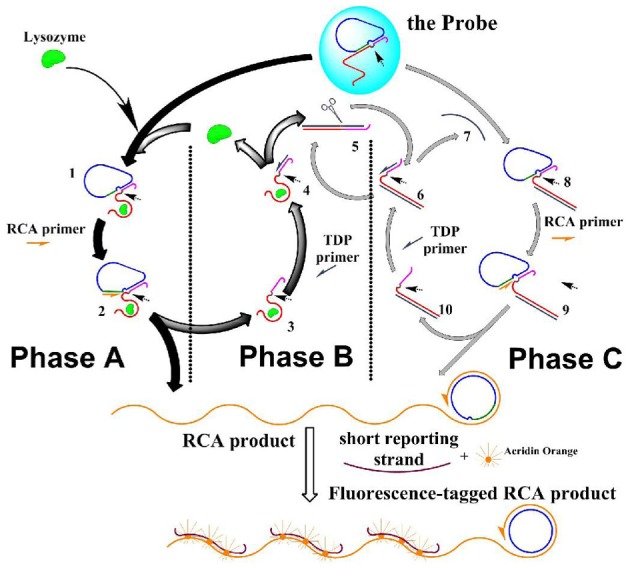
**The scheme of the DNA molecule machine with three phases.** Half-arrows on the strands indicate the extending direction of the polymization. Small arrows in the structure of the species indicate the potential nicking site.

The reactions (“movements”) of the DNA molecular machine were designed to run in three phases that were in accordance to the two functions: the whole machine centered on the triggered RCA (phase A) to bring about the morphology change so that the machine can be visualized; and the RCA was then repeated due to TDP (phase B) and TDP-repeated RCA enhanced by NPC (phase C) to provide multi-phase amplification. All these phases and reaction steps are shown in Scheme 1.

Phase A: the molecular machine started with phase A, in which RCA was triggered by lysozyme. This phase started with the aptameric recognition of lysozyme, and resulted in RCA that produced long strand with periodic repeated sequences. The process: lysozyme interacted with the aptamer strand, “turning on” the RCA switch: due to the high affinity between lysozyme and its aptamer, the later was separated from the “circle template” at the RCA switch site, and bound to lysozyme instead; thus the RCA switch site was exposed (1 in Scheme 1). RCA primer could thus bind to this empty site (forming 2 in Scheme 1), and started a rolling circle polymerization based on the circle template in the presence of the DNA polymerase, and produced two results: a long strand with periodic repeated sequences was produced, and lysozyme-aptamer complex (5 in Scheme 1) was displaced by the extending long RCA product strand and separated from the “circle template.” This forms the starting point of phase B.

Phase B: lysozyme was recycled in target-displacement polymerization (TDP), thus RCA in phase A could be repeated. This phase started with the binding of TDP primer to the TDP switch site (an overhang in the complex of lysozyme and its aptamer), and resulted in the release of lysozyme from its aptamer. The process: at the end of phase A, TDP switch was turned on: the TDP switch site was exposed on the lysozyme-aptamer complex (in 4 Scheme 1); TDP primer was then able to bind this site and initiated DNA polymerization on the aptamer template; the polymerization produced two results: (a) the bound lysozyme was displaced and released into the solution, which could then interact with another probe to start another round of RCA, thus phase A was repeated; and (b) The polymerization produced a dsDNA containing the aptamer template (5 in Scheme 1), and an actual Nb.BbvCI nicking site was formed on this duplex, which would start phase C.

Phase C: a Nicking-polymerization cycle (NPC) took place based on Phase B, this caused repetitive production of an ssDNA that mimicked lysozyme, which, as a starter, initiated a reaction cycle that mimicking phase A and phase B, causing the RCA to be repeated with more turnovers. This phase started with the repetitive production of an ssDNA strand in NPC, and resulted in the multiplied RCA. The process: the dsDNA (repetitively produced in phase B, (5 in Scheme 1) had an actual nicking site on it, turning on the NPC switch: the dsDNA was nicked in the presence of NEase Nt.BbvCI (6 in Scheme 1); from the nicked site, a nicking-polymerization cycle (NPC) was started, and repetitively produced a single stranded DNA (7 in Scheme 1). This ssDNA is complementary to the aptamer sequence in the aptamer strand, just analogous to lysozyme can bind to this strand. Thus, this ssDNA acted in the molecular machine just as an analog to the lysozyme—causing a series of reactions just as lysozyme does in phase A and phase B, and at last this ssDNA itself be recovered: it bound to the aptamer in the probe, and TCA switch was “turned on” as in phase A, caused the breakdown of the substrate probe (8 in Scheme 1), RCA was then initiated (9 in Scheme 1); and then, a duplex of the aptamer strand and the ssDNA was formed, on which the TDP switch was “turned on,” causing a strand-displacement polymerization (10 in Scheme 1). Thus, more RCA are conducted without more lysozyme inputted, even without the turnover of the lysozyme itself.

TIRFM (carried out on a Leica AM TIRF MC apparatus) was employed to visualize the reaction change and signal amplification in the molecular machine. Concise procedures: After reactions, the mixture was treated with short reporting strands, which were complementary to the expected long-strand RCA products, and then stained by SYBR Green (SBSBIO, Beijing, P.R. China), and placed on the well of glass-bottom petri dish for TIRFM, and the DNA species were allowed to absorb onto the surface of the glass bottom under pH 4.0 according to previous studies on the affect of pH on the absorption of DNA on glass surface (Li, 2009; Yin, 2009). The reaction system under different raw feed configurations and different reaction conditions were interrogated with TIRFM to study the progress of the reaction.

### Demonstration of the morphology change before and after the reaction

Examination of the reaction products: it was clearly shown that, after sufficient reaction (see supplementary materials for detailed Procedures), thread-like fluorescent objects appears with the width less than 0.5 μm; these threads were not visible under the transmitted light mode (Data not shown), indicating these fluorescent objects were of sizes of nanometer scale. Furthermore, treating the samples with DNase I (an endonuclease that degrades any poly-deoxynucleotides, single-stranded or double-stranded) eliminated all the fluorescent objects (Data not shown), verifying that the fluorescent dots and thread-like objects are all of DNA nature. These can be attributed to the long-chain RCA products, which produce fluorescence after hybridizing with the short reporting strands to form long-chain DNA with double-stranded segments, which were then intercalated with the pigment SYBR Green.

Examination of the raw materials before the reaction: on the other hand, in the blank status, When the mixture of the raw feeds (i.e., the probe and the two primers) was examined, fluorescent dots can be seen all over the view field. only dispersed fluorescence dots can be seen. These can only be attributed to the DNA probe in the raw feeds. Comparison: thus, in TIRFM the probe and the RCA products can be discriminated, and the transformation from the fluorescent dots to the fluorescent threads can be clearly exhibited, which is also the exhibition of the reported molecular machine and the signal amplification in it.

### The products of the molecular machine at different phases

**Figure 1:**
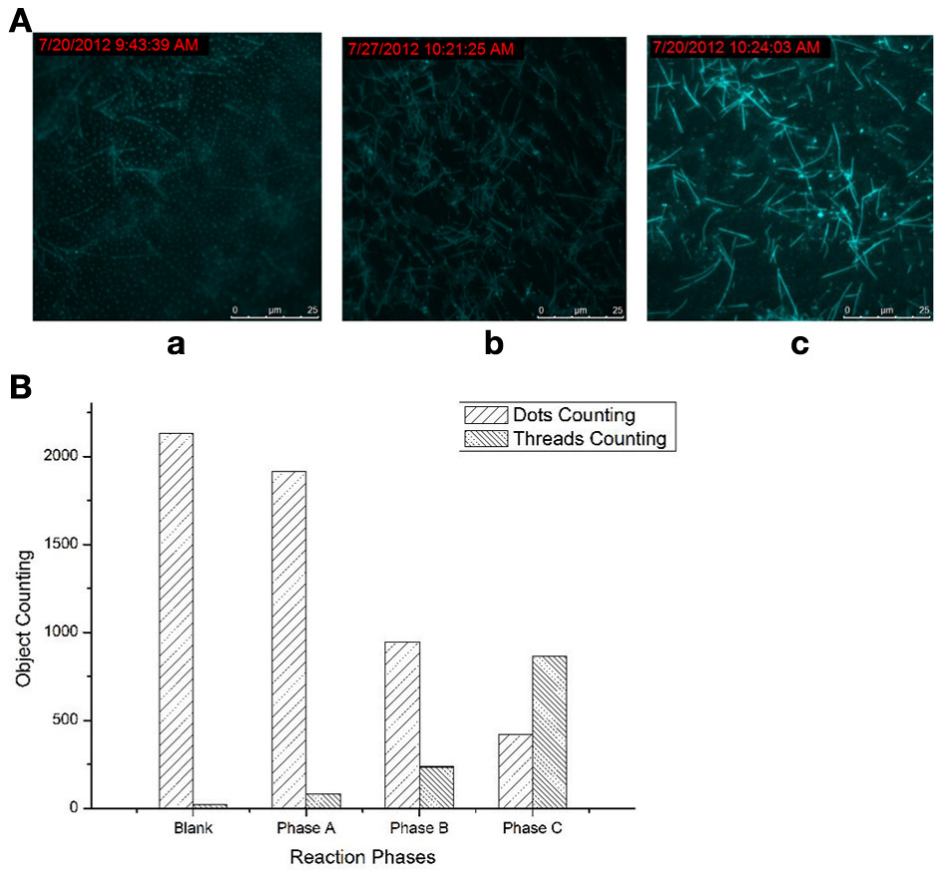
**Products of the molecular machine at three phases. (A)** TIRFM visualization: (a) Phase A, simple RCA (rolling-circle amplification); (b) Phase B, RCA cycled and amplified by TDP (target-displacement polymerization); (c) Phase C, the full molecular machine, RCA cycled by TDP which was further cycled and amplified by NPC (nicking-polymerization cycle). **(B)** Counting results of the dots and thread-like objects.

Difference between the three phases: it can be clearly shown that in phase A, fluorescence dots widely and densely dominated in the whole vision field (Figure 1Aa), with a few fluorescent threads dispersed in the matrix of the bright dots. For the products of phase B (Figure 1Ab), the fluorescent dots apparently became scarce, and fluorescent lines begins to appear; and in phase C (Figure 1Ac), all the fluorescent dots nearly vanished, and the fluorescent threads occupied the whole vision field. Thus, the three phases of the molecular machine can be clearly distinguished: from the majority fluorescent dots to the majority of fluorescent threads; and the density of the fluorescent threads increased through the three phases.

### Quantitative statistics of the fluorescence objects

The fluorescent dots and threads are sorted and counted by image analysis on these visualizations using ImagePro Plus, in which the dots and threads can be easily discriminated by area and aspect ratio. The counting was conducted based on the unit view field of 100 × 100 μm^2^ (under 100 × objective). The results of sorting and counting were shown in Figure 1B. Every counting results is an average of at least ten images obtained under the same condition.

It was more clearly shown by the counting results—the significant increasing in the threads counting along with the obvious reducing in the dots counting through the phases. Thus, the transformation from the raw feeds (fluorescent dots) to the RCA products (fluorescent threads) can be both visually and quantificationly exhibited. When the higher phases were opened up, more such transformations, i.e., reactions, or the signal amplifications, can be directly observed.

### The depenence between the products producing, probe consuming and the lysozyme concentration

**Figure 2:**
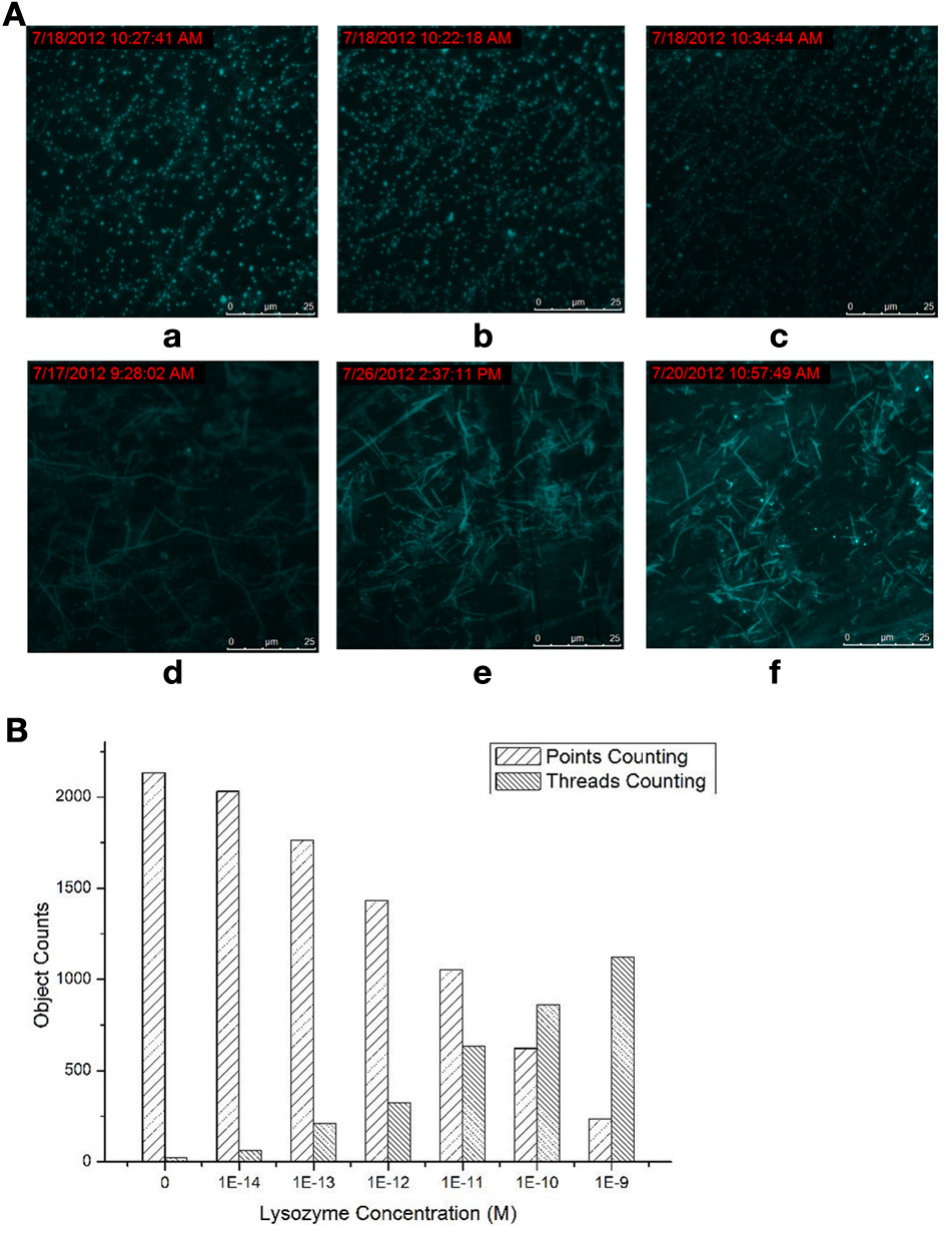
**Products of the molecular machine when different lysozyme concentrations were applied. (A)** TIRFM visualization: (a) blank, (b) 10^−13^ M (c) 10^−12^ M (d) 10^−11^ M (e) 10^−10^ M (f) 10^−9^ M; **(B)** Counting results of the dots and thread-like objects.

The dependence between the products producing, probe consuming and the lysozyme concentration were also interrogated (Figure 2). The TIRF images of the fluorescence-tagged reaction mixtures were shown in Figure 2A, and the quantitative analysis data were summarized in Figure 2B. When the lysozyme concentration was varied, in accordance with the signal increasing along with the increasing of the lysozyme concentration (Figures S3 and S4 in Supplementary Materials), TIRFM clearly showed the increasing of the thread-like fluorescent objects and the decreasing of the dot-like objectives. Thus, the progress of the DNA reaction system along with the lysozyme (target) concentration can be visually tracked with TIRF.

### The progress of the molecular machine along with the reaction time

To track the progress of the molecular machine along with the reaction time, the changes of the products producing and probe consuming along with the increasing of the reaction time were interrogated (Figure 3). The TIRF images of the fluorescence-tagged reaction mixtures were shown in Figure 3A, and the quantitative analysis data were summarized in Figure 3B. Along with the reaction time, in accordance with the fluorescence signal increasement (Figure S2C in Supplementary Materials), TIRFM clearly showed the increasing of the thread-like fluorescent objects (products) and the decreasing of the dot-like objectives (raw materials). Thus, the progress of the DNA reaction system along with reaction time can be visually tracked with TIRF.

**Figure 3A:**
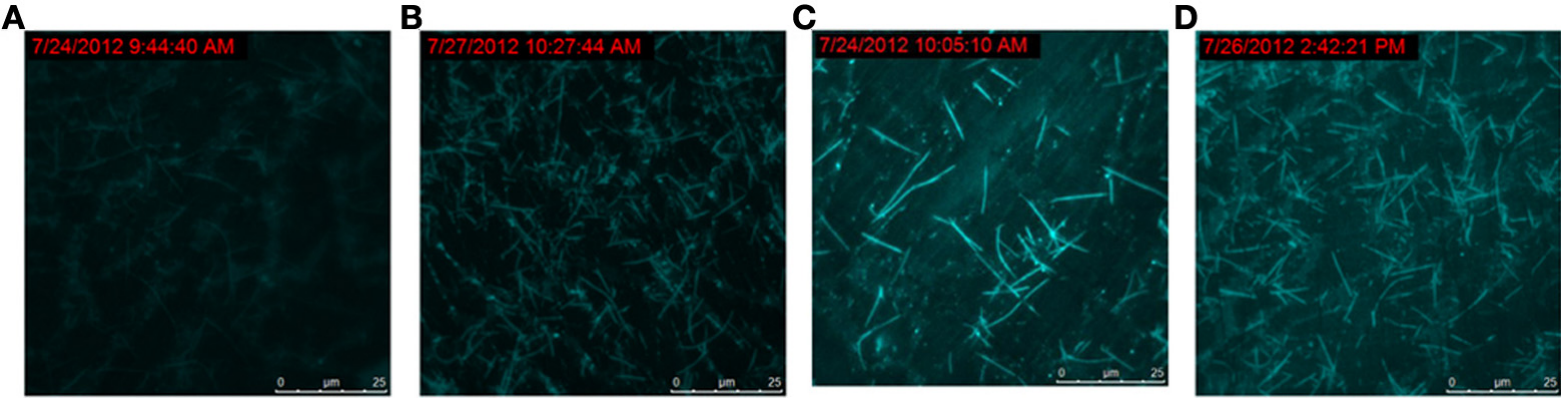
**Figure 3A. Products of the molecular machine when different reaction time. (A)** 0.5h, **(B)** 1.0h, **(C)** 2.0h, **(D)** 3.0h.

**Figure 3B:**
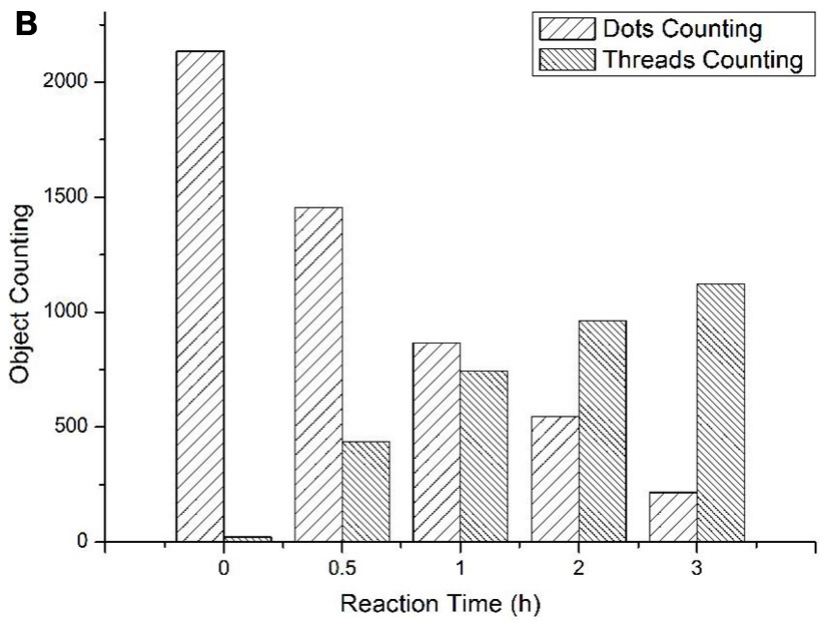
**Figure 3B. Products of the molecular machine when different reaction time. (A)** TIRFM visualization. (a) 0.5h, (b) 1.0h (c) 2.0h (d) 3.0h; **(B)** Counting results of the dots and thread-like objects.

## Conclusions

In this paper, the amplification in a DNA molecular machine was directly visualized via TIRF. As a proof of principle, a molecular machine involving three amplification layers was established as the model process to exhibit the capability of TIRF to visualize a biochemical process at the single-molecule level. The movements of the molecular machine featured the triggered and repeated RCA based on a “blocked” circular template. Initiated by lysozyme (the input): the circular template was “unblocked” to start RCA, producing long-chain tandem RCA products, and at the same time, causing TDP and NPC reactions in cascades, which in turn repeated RCA, multiplying the RCA product, bringing forth signal amplification (relative to the original inputted lysozyme). The molecular machine was interrogated using traditional fluorescence quantification approach and TIRF direct visualization approach. Through TIRF approach, the transformation of the raw feeds (dot-like species) to the thread-like species (RCA products) can be clearly revealed at the molecule level; the dependency of the aforementioned morphology conversion on the trigger (lysozyme) can be verified by the increasing of the conversion ratio along with the increasing of lysozyme concentration; the progressive amplification from phase to phase can be exhibited through the prominent increasing in the conversion of thread-like objectives through the phases; and the proceeding of the reaction can be visualized through the increasing conversion through time with the increasing of the concentration of the trigger (lysozyme). Overall, it was demonstrated that TIRF is an efficient approach to visualize the biochemical processes at single-molecule level.

## Materials and methods

### General materials

The Klenow fragment of *E. coli* DNA polymerase I (10 U μL^−1^, denoted as “Klenow” for short), reaction buffer for Klenow (denoted as “Klenow buffer” for short), and the mixture of 4 dNTPs (2.5 mM for each component) were purchased from Fermentas Inc. (Glen Burnie, ML, USA). Nicking endonuclease Nb.BbvCI (10 IU μL^−1^, potential nicking site: CCTCA▲GC) and its buffer (NEBuffer 2) were obtained from New England Biolabs Inc. (Ipswich, MA, USA). The carboxyl-modified magnetic beads (Affimag PSC 3422, particle diameter: 1.0–2.0 μm, particle content in suspension: 10 mg mL^−1^, denoted as “MB” for short) were obtained from BaseLine ChromaTech Research Center (Tianjin, China). Imidazole, N-hydroxysuccinimide (NHS), 1-ethyl-3-(3-dimethylaminopropyl) carbodiimide (EDAC) and tris(hydroxymethyl)aminomethane (tris) were purchased from Sigma-Aldrich Co. LLC. (St. Louis, MI, USA).

### DNA strands and probes

6 DNA strands were used in this molecular machine (for detailed sequences see the Supporting Information) to build the probe complex and 2 primer strands (primer 1 and 2). The probe complex (Scheme S1 in Supplementary Materials) is assembled using an ssDNA strand containing lysozyme aptamer segment (“aptamer strand”), a circular strand that is partially complementary to the aptamer strand and a “binding” strand (see Scheme S1 in supporting information for detailed structure of probe complex). Two other strands were used as the primers (primer 1 and primer 2).

For the detailed sequences of the strands, the detailed structure and preparation procedure of the probe complex, please refer to the experimental section in the Supporting Information.

### Operation of the molecular machine

When the molecular machine was operated for fluorescence measurement, typically, the probe complex, primers 1 and 2, Klenow, Nb.BbvCI and dNTP were mixed in a 1-mL Eppendorf tube, and 10 μL lysozyme solution of specific concentration was added to form a reaction mixture containing the probe complex (5.0 × 10^−8^ μM), primer 1 (0.50 μM), primer 2 (0.50 μM), NEBuffer 2, dNTP mixture (0.25 mM for each component), Klenow (0.20 IU μL^−1^), Nb.BbvCI (0.12 IU μL^−1^) and lysozyme of certain concentration. The mixture was incubated at 37°C for 3 h, and then magnetically separated. Supernatant (circa 120 μL) was collected, and the MB residue was washed three times with PBS buffer (pH 9.0), and all the washing solution was merged into the supernatant to form a solution (about 700 μL).

The pH of the solution was adjusted to around 4, and 50 μL of the solution is dropped on the bottom-surface of the glass-bottom dish, and the fluorescence strand was added and the fluorescence marking was allowed to proceed for 30 min, and then was recorded using TIRFM using 100× objective with the numeric aperture 1.46.

In comparison experiments, the probes, primers, and enzymes were fed with different recipes. The detailed procedures are elucidated in the Supporting Information.

## Conflict of interest statement

The authors declare that the research was conducted in the absence of any commercial or financial relationships that could be construed as a potential conflict of interest.
