# Supplementary material for: Direct visualization of the reaction transformation and signal amplification in a DNA molecular machine with total internal reflection fluorescence microscopy
Source: Front Chem. 2013 Oct 31;1:23. doi: 10.3389/fchem.2013.00023 (PMC3982516; doi:10.3389/fchem.2013.00023)
Supplement: Supplementary file 1 [file DataSheet1.PDF]

Supporting Information

Direct Visualization of Amplification in a DNA Molecular Machine with Total Internal Reflection Fluorescence Microscopy

*Rui Ren, Haiyan Wang, Mingyin Wang, Rui Liu and Shusheng Zhang**

Key Laboratory of Biochemical Analysis, Ministry of Education, College of Chemistry and Molecular Engineering,

Qingdao University of Science and Technology, Qingdao 266042

Table of Contents

S1. Experimental Section ……………………………………………………………………S2

S2. The details of the structure of the probe …………..…………………………….…..…S5

S3. The detailed reaction process of the molecular machine…………………………..…………S6

S4. Optimization of reaction conditions…………………………………….……………………S11

S5. Fluorescence readouts of different phases……………………………….……………………S12

S6. Protocols implementing the TIRFM visualization S

S7. Additional Results of TIRFM visualization S

S8. Selectivity ……………………………………………………………………………………S14

S9. Determination of Real Samples……………..………………………………………..……S14

References…………………………………………………………………………………….S14

S1. Experimental Section

S1.1. DNA sequences. The DNA oligomers used in this study were synthesized by SBS Genetech Co. Ltd. (Beijing, China). Their detailed sequences are listed in Table S1.

Table S1. DNA Oligomers Sequences

Strand Names	**Sequence**
**Circle template**	5'-CCT CTA AGT AAC TCT GAG GAT TAG GAT TAG GAG GAG GAG GTA GGG CGG CTT CGG ATT TTT TTT TTT TGA CAC CAT TAC C -3' (purchased in the closed circle form)
**Aptamer template**	5'- ATC TAC GAA TTC ATC AGG GCT AAA GAG TGC AGA GTT ACT TAG AGG CCT CA▲G CGG TAA TGG TGT CTT - 3'
**Binding Strand**	5'-NH$_2$-AAA CCC CCT CCT CCT CCT AAT CCT AAT CCT CCC CC-3',
**RCA primer**	5'- AGA GTT ACT TAG AGG -3'
**TRP primer**	5'-GAC ACC ATT ACC-3'
**Fluorescence signal strand**	5'-TAMRA- TTT TAG GGC GGC TTC GGA TAA -3'
**Signal binding strand**	5'-NH$_2$-AAA AAA AAA TTC CGA AGC CGC GG-3'

* **Note:**
1) The segments, in which the letters are rendered with same font color and same background color, are identical or complementary.
2) The triangle ▲ indicates the potential nicking point of Nb.BbvCI, which means, it would lead to the formation of an actual Nb.BbvCI nicking point on its complementary strand upon hybridization.

7 oligonucleotide strands were used in the construction of the molecular machine. A **circle template** (obtained in the closed circle form) is complementary to an **aptamer template** in 2 separated segments, which were separated by a potential nicking site of NEase Nb.BbvCI on the **aptamer template**. **The aptamer strand** contains the lysozyme aptamer sequence and the potential nicking site of Nb.BbvCI (an actual Nb.BbvCI nicking site could be activated on its complementary sequence upon hybridization). To ensure no excess aptamer strand was left in the molecular machine (which would bind lysozyme but do not initialization the amplification reactions and thus bring about a false negative response) after the preparation of the probe, a "binding strand" was utilized to attach the complex of the circle template and the aptamer template to the magnetic beads, thus the complex can be easily separated and purified. Two short primers are used for polymerization: **RCA primer** is

complementary to part of the circle template, and, as its name indicates, can initialize RCA; and TDP **primer** is complementary to part of **aptamer strand**, which initialize TDP.

A reporting probe is used to convert the RCA product into fluorescence readout (for traditional approach) and image (for TIRFM visualization approach), in which a fluorescence-tagged strand was bound to the magnetic bead by a binding strand. The reporting probe consisted by a TAMRA-tagged “signal” strand and a “signal binding” strand that was partially complementary to it. The later binds the former on the magnetic bead; and when any RCA product was generated in the molecular machine, it would release the signal strand from the reporting probe through strand-displacement hybridization, since the complementary part between the signal strand and the signal binding strand is shorter than that between the RCA product and the signal strand.

S1.2. Other materials.

Klenow fragment of *E. coli* DNA polymerase I (10 IU μL^{-1}, denoted as “Klenow” for short), the buffer for Klenow-catalyzed polymerization (denoted as “Klenow buffer” for short) and mixture of four dNTPs (2.5 mM for each component) were purchased from Fermantas Inc. Nicking endonuclease Nb.BbvCI (nicking site: GC▲TGAGG) and its buffer NEBuffer 2 were obtained from New England Biolabs. Other materials are purchased from Sigma-Aldrich Inc. The molecular beads (MBs) used in this work is the model PSC-3412 provided by Baseline ChromaTech (Tianjin, P. R. China), which consisted of a polystyrene core and a Fe_3O_4 shell, with the particle diameter of 1-2 μm; the MBs is supplied in a suspension of 5.0 mg mL^{-1} content.

Buffer solutions:

Imidazole-HCl solution was prepared by mixing 0.1 mol HCl with 0.1 M imidazole solution to reach a pH of 6.8.

TAE (Tris-acetate EDTA) buffer contains 40 mM tris(hydroxymethyl)aminomethane (tris), 2 mM EDTA and 20 mM acetic adic, and the pH was adjusted to 7.9.

PBS (phosphate buffered saline) buffer contains 137 mM NaCl, 2.7 mM KCl, 8.1 mM Na_2HPO_4 and 1.76 mM KH_2PO_4. The newly prepared PBS has a pH of 7.4, which can be adjusted with HCl or NaOH if other pH is desired.

Klenow buffer (10×) was provided by Fermentas Inc. It contains 500 mM Tris-HCl (pH 8.0 at 25 °C), 500 mM NaCl, 100 mM $MgCl_2$ and 10 mM DTT, and was diluted 10-fold when use.

NEBuffer 2 (10×) was provided by New England Labs. It contains 100 mM Tris-HCl (pH 8.0 at 25°C), 50 mM $MgCl_2$ and 10 mM DTT, and was diluted 10-fold when use.

S1.3. Instruments

TIRFM imaging was carried out on a Leica AM TIRF MC unit with the exciting wavelength set at 488 nm (for GoldView) and with a magnification ratio of 1000 ×.

Fluorescence spectra were recorded on a Hitachi F-4600 fluorescence spectrophotometer with a 1-cm silica cuvette. The excitation wavelength was set to 535 nm, and the emission was measured at 575 nm (for TAMRA fluorescent tag, or 5-carboxytetramethylrhodamine).

Non-denaturing polyacrylamide gel electrophoresis (PAGE) was carried out on a DYCZ-24DN electrophoresis cell with DYY-6C power supply (both from Beijing Liuyi Instruments, Beijing, China), and the PAGE patterns were imaged on a WD-9413B gel imaging system (Beijing Liuyi Instruments, Beijing, China). The PAGE was generally run at 140 V for 2 h.

S1.4. Preparation of the substrate probe.

To prepare the **substrate probe**, the **binding strand** was first attached to the MBs through the amide bond. In detail, the MBs were washed three times with 0.1 M imidazole-HCl solution (pH 6.8), and re-dispersed in TAE buffer to form a dispersion with a MB content of 50 mg mL^{-1}. The dispersion was mixed with EDAC solution (0.1 M) with a volume ratio of 3:10, and then incubated at 37 °C for 60 min to activate the carboxyl groups on the MBs.

150 μL solution of **binding strand** in TAE (10^{-7} M) was mixed with 200 μL activated MB suspension, and the linkage reaction was allowed to proceed at 37 °C for 12 h, after which the unattached DNA strands were removed by magnetic separation. The MB residues (with **binding strand** attached) were then re-dispersed in 200 μL TAE buffer to retain the original content of 5.0 mg mL^{-1}, in which the binding strand got a concentration of about 7×10^{-8} M。

On the other hand, 110 μL of **aptamer template** (10^{-7} M) and 110 μL **circle template** (10^{-7} M) was mixed and heated to 90 °C, and then slowly cooled to the room temperature in approximate 2 h to ensure that the two strands could thoroughly hybridize with each other. Then 200 μL solution of hybridization product was mixed with 150 μL suspension of the magnetic beads attached with the **binding strand**, and the mixture was then incubated for 60 min at 30 °C. The prepared probes were then washed thrice with PBS, and magnetically separated. The residue was re-dispersed in TAE to form a 500 μL suspension in which the concentration of the circle-aptamer complex is estimated to be

3×10^{-8} M. The suspension was maintained at a temperature < 4 °C.

To prepare the reporting probe, the magnetic beads were washed and activated using the same procedure as described above. The **signal binding strand** was attached to MBs, and then hybridized with the **signal strand** using the same conditions and procedures as described above; except that these two strands were added at much higher concentrations, which resulted a final probe concentration of about 10^{-6} M in the resulted reporting probe suspension.

S1.6. Procedure to operate the molecular machine.

When the molecular machine was operated for TIRFM visualization, typically, the suspension of **substrate probe** (30 μL), the two primers (3 μL each), dNTP (10 μL), Klenow (1.5 μL), Nb.BbvCI (1.0 μL), NEBuffer 2 (10 ×, 10 μL) and 10 μL lysozyme solution or diluted cell lysate were mixed in a 1-mL Eppendorf tube to form a reaction mixture containing the probe (2×10^{-8} M), two primers (6.0×10^{-7} M each), NEBuffer 2 (1 ×), dNTP mixture (0.25 mM for each component), Klenow (0.15 IU μL^{-1}), Nb.BbvCI (0.10 IU μL^{-1}) and lysozyme of certain concentration. The mixture was sonicated to ensure all the components were mixed sufficiently, and then incubated at 30 °C for 3 h, and then magnetically separated.

When the molecular machine was operated for fluorescence measurement, typically, the suspension of **substrate probe** (80 μL), the two primers (3 μL each), dNTP (10 μL), Klenow (1.5 μL), Nb.BbvCI (1.0 μL), NEBuffer 2 (10 ×, 10 μL) and 10 μL lysozyme solution or diluted cell lysate were mixed in a 1-mL Eppendorf tube to form a reaction mixture containing the probe (2×10^{-8} M), two primers (6.0×10^{-7} M each), NEBuffer 2 (1 ×), dNTP mixture (0.25 mM for each component), Klenow (0.15 IU μL^{-1}), Nb.BbvCI (0.10 IU μL^{-1}) and lysozyme of certain concentration. The mixture was sonicated to ensure all the components were mixed sufficiently, and then incubated at 30 °C for 3 h, and then magnetically separated.

S1.7. Fluorescence measurement of the reaction products.

Supernatant (circa 90 μL) was collected and mixed with 60 μL suspension of the MBs attached with the **reporting probe**. The mixture was heated to 55 °C, and then slowly cooled down to allowed to 30 °C, and then allowed to stand at 30 °C for 60 min, and then magnetically separated. Supernatant (circa 150 μL) was collected and the MB residue was washed three times with PBS buffer (pH 9.0), and all the washing solution was merged into the supernatant to form a solution (about 700μL) for the

fluorescence measurement.

S2. The details of the structure of the substrate probe.

The detailed structure of the “substrate probe” was shown in scheme S1. Two primers, RCA primer and TDP primer were used along with the probe to initiate the polymerizations. The main part of the probe is a complex of a circularized DNA template and an aptamer template that annealed to each other to form three reaction switches.

The first switch (for RCA) was a duplex segment that formed by the green segment in the circle template and its complementary segment in the aptamer template. The green segment is complementary to the RCA primer, and its complementary segment (whose sequence is the same as the RCA primer) is part of the aptamer sequence of lysozyme. In intact probe, the binding between the two segments blocked the attaching of the RCA primer; but when lysozyme bound to its aptamer, the binding between the two segments would be dissociated, and RCA primer would be allowed to attach to the green segment in the circle template and initiate RCA.

The second switch (for TDP) was a duplex segment that formed by the magenta segment in the aptamer template and its complementary segment in the circle template. The magenta segment is complementary to the TDP primer, and its complementary segment (whose sequence is the same as the RCA primer) is *not* a part of the aptamer sequence of lysozyme. In intact probe, the binding between the two segments blocked the attaching of the TDP primer; but during the RCA in the first phase, the extending long strand would dissociate the binding between the two segments by strand displacement, and TDP primer would be allowed to attach to the magenta segment in the circle template and initiate TDP.

The third switch is a potential nicking site of nicking endonuclease (NEase) that was placed on the aptamer strand between the two annealed segments, shown in brown**.** In the intact probe, the nicking site exists in single strand form and did not work. During TDP that using the aptamer template as the template, an actual nicking site would be formed, and was nicked to initiate NPC.

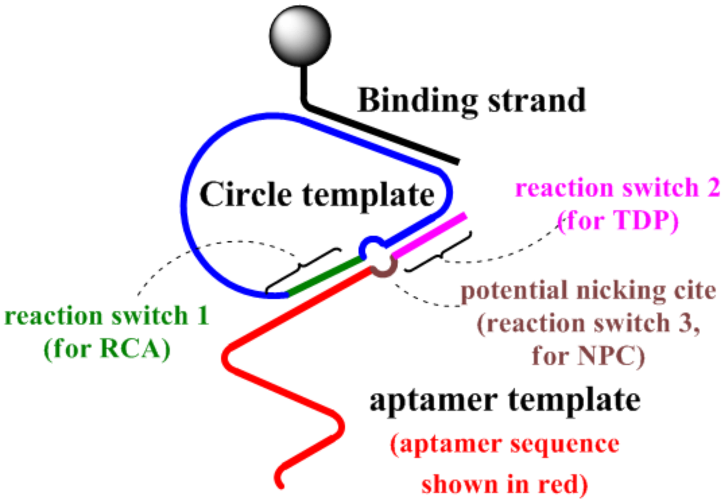

Scheme S1 Details of the structure of the substrate probe

S3. The detailed reaction process.

The whole molecular machine originated from phase A, triggered RCA. This phase started with the aptameric recognition of lysozyme by the aptamer sequence in the aptamer strand, and resulted in RCA that produces the long tandem strand. The binding of lysozyme, the target, to the aptamer sequence (red segment in scheme S1) in the aptamer strand caused the partial dissociation of the later from the green segment in circle template, forming an "opened substrate probe" (**1**); the first reaction switch was turned on: the green segment in circle template was exposed, to which the RCA primer was attached, forming an "opened substrate probe with RCA primer" (**2**), thus RCA was started; in the first round of RCA, the new strand extended from the RCA primer along the circle template, thus the complex of lysozyme and its aptamer (**3**) was displaced. The magenta overhang in the aptamer template was exposed, which would enable phase B.

Phase B: TDP-enhanced RCA. This phase starts with the binding of TDP primer to the magenta overhang in the aptamer strand that was exposed due to the strand-displacement in the RCA, and resulted in the repeating of the RCA due to the repetitive releasing of lysozyme. After the attaching of the TDP primer to the magenta overhang (**4**), DNA polymerization was initiated along the aptamer template, during which the bound lysozyme was displaced and released, and was then able to be feedback to phase A to break down another substrate probe for another round of RCA, and RCA can be repeated. In the polymerization, a dsDNA using the aptamer template (**5**)

was formed, and an actual Nb.BbvCI nicking site was formed due to the formation of the duplex, forming the stimulus of phase C.

Phase C, NPC-enhanced TDP-enhanced RCA. This phase was initialized by the nicking of a double-stranded DNA (**5**) that was formed in the TDP in phase B, and also resulted in the extra repetition of RCA. The nicking in the dsDNA (**5**) produced the nicking product (**6**), in which the nick acted as the starting point of a polymerization, in which a single stranded DNA (**7**) was repetitively produced, which is complementary to the aptamer strand roughly in the aptamer segment, which can be dubbed "lysozyme equivalent ssDNA". It is so named for that, this ssDNA binds to the lysozyme aptamer (the red segment in the aptamer template) just as lysozyme, and can also be displaced in the polymerization; thus cycling reactions in analogue to those in phase A and phase B occurred: the binding of ssDNA (7) to the aptamer sequence in the aptamer strand caused the partial dissociation of the later from the green segment in circle template, forming an "opened substrate probe" (**8**); the first reaction switch was turned on: the green segment in circle template was exposed, to which the RCA primer was attached, forming an "opened substrate probe with RCA primer" (**9**), thus RCA was started; in the first round of RCA, the new strand extended from the RCA primer along the circle template, thus the dsDNA of ssDNA (7) and the aptamer template (**10**) was displaced. The magenta overhang in the aptamer template was exposed, to which the TDP primer was attached, and (**6**) is regenerate. Polymerization took place on (6) and resulted in (5) and (7); and another round of cycle could began.

S4. Procedures to carrying out TIRF

Since the principle of TIRFM required that only the moleculars laid within 200 nm from the surface of coverglass can be observed. Thus the visualization using TIRFM involved depositing the sample molecules on the surface of the cover glass. This was done by treating the sample with the buffer at pH 4, which causes the the DNA strands deposited on the surface of the coverslip

S5. Optimization of reaction conditions

Various factors affected the polymerization and nicking processes, and thus produced a final effect on the amplification efficiency of the molecular machine.

The optimum pH, indicated by the user manual for enzymes used in this study, *E. Coli* DNA Polymerase I Klenow fragment ("Klenow" for short), and the nicking endonuclease, Nb.BbvCI. vary

slightly (pH 7.5 for Klenow and pH 7.9 for Nb.BbvCI). It was proven that choosing either of these two pHs did not lead to observable difference in the results (data not shown). In this study, Thus, the variable conditions, i.e. the time duration and the content of the 2 enzymes, were examined as described below.

The reaction time was tested in the range from 0.5 h to 4 h using the procedures depicted in S1.6 and S1.7. The fluorescent response reached a platform after a reaction time of 3 h, which was then chosen as the optimal time (Figure S2A).

The individual effects of Klenow content and Nb.BbcVI content on the output of the molecular machine were also examined. Using the procedures depicted in S1.6 and S1.7, the Klenow concentration was examined from 0 to 0.30 IU μL^{-1}, and the optimum value was found to be 0.15 IU μL^{-1} (Figure S2B); and the Nb.BbvCI concentration was examined from 0 to 0.30 IU μL^{-1}, and the optimum value was found to be 0.10 IU μL^{-1} (Figure S2C). The optimum enzyme concentrations were then determined.

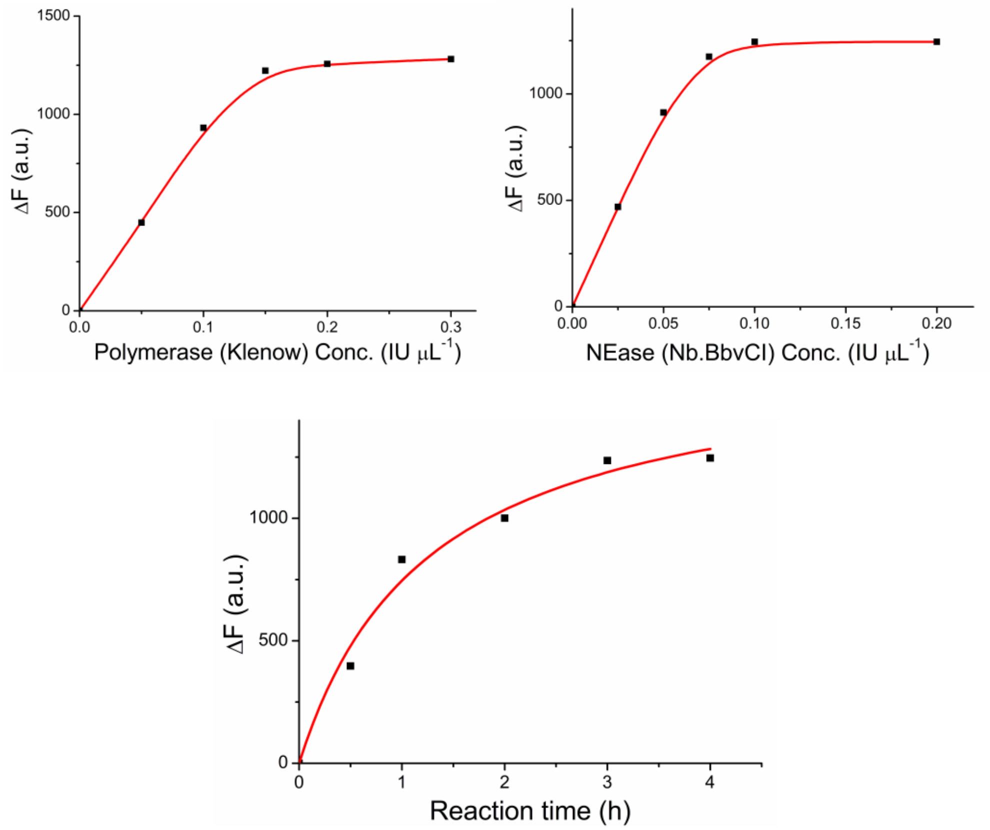

Figure S2. Optimum reaction conditions. (A) Concentration of *E. Coli* DNA polymerase I Klenow fragement; (B) Concentration of NEase Nb.BbvCI; (C) Reaction time.

6 Fluorescence results and calibration curves

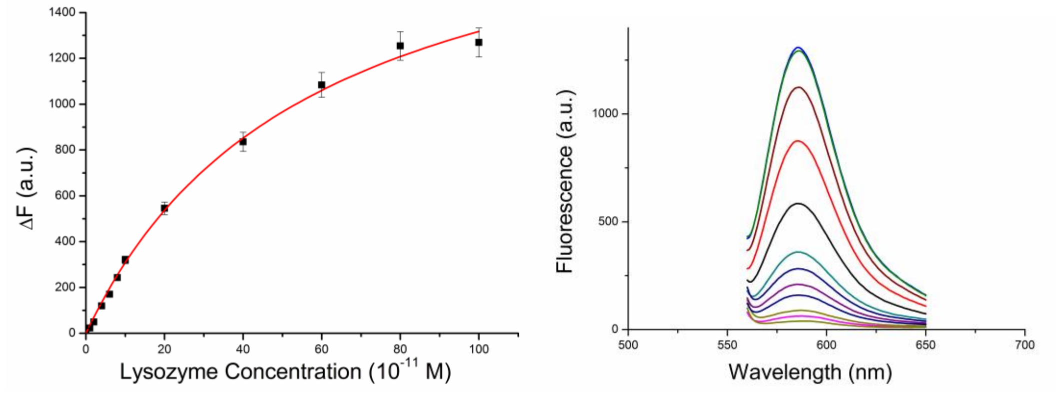

Figure S3 Fluorescence Curves of Phase A. From bottom to top: blank; 1.0×10^{-11} M; 2.0×10^{-11} M; 4.0×10^{-11} M; 6.0×10^{-11} M; 8.0×10^{-11} M; 1.0×10^{-10} M; 2.0×10^{-10} M; 4.0×10^{-10} M; 6.0×10^{-10} M; 8.0×10^{-10} M; 1.0×10^{-9} M.

The signal amplification in the reported complex can be exhibited by comparing its sensitivity performance with those of different phases. In **phase A** (the reactions in solid wide arrows in the left of Scheme 1), the calibration curve, i.e. the curve of fluorescence response against the concentration of lysozyme, is shown in Figure 2. The fluorescence responses (figure S4 in ESI) approximately formed a Michaelis relation against the concentration of lysozyme in the range from 1.0×10^{-11} M to 1.0×10^{-9} M, with a fitting equation of $\Delta F = 2071.7\ c/(57.22 + c)$ (ΔF, any unit; c, 10^{-11} M, Figure 2). A detection limit of 3.8×10^{-12} M is obtained according to the 3σ rule. This is the sensitivity of the simple RCA itself, which is two orders of magnitude lower than that of the whole molecular machine.

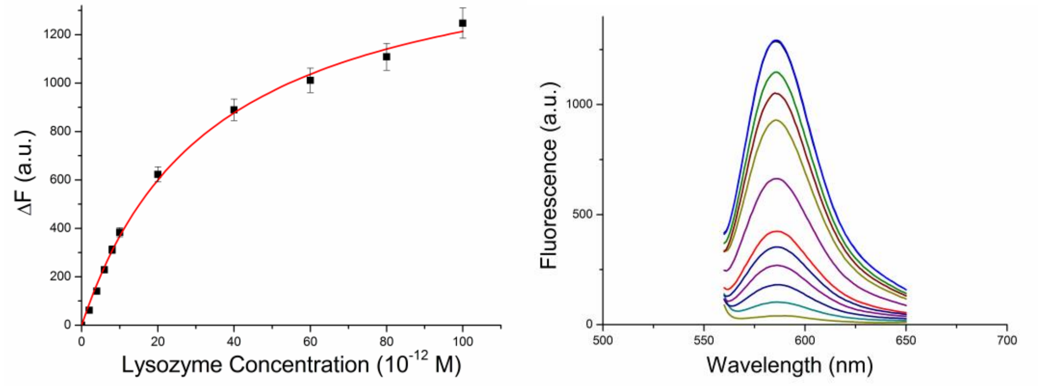

Figure S4 Fluorescence Curves of Phase 2. From bottom to top: blank; 2.0×10^{-12} M; 4.0×10^{-12} M; 6.0×10^{-12} M; 8.0×10^{-12} M; 1.0×10^{-11} M; 2.0×10^{-11} M; 4.0×10^{-11} M; 6.0×10^{-11} M; 8.0×10^{-11} M; 1.0×10^{-10} M

In **phase B**, the calibration curve gave a Michaelis relation between fluorescence response (figure S5 in ESI) and the concentration of lysozyme in the range from 2.0×10^{-12} M to 1.0×10^{-10} M, with a fitting equation of $\Delta F = 1629.9\ c/(34.89 + c)$ (ΔF, any unit; c, 10^{-12} M Figure 3). The detection limit was found to be 2.7×10^{-13} M (3σ rule), one order of magnitude lower than that in **phase A**.

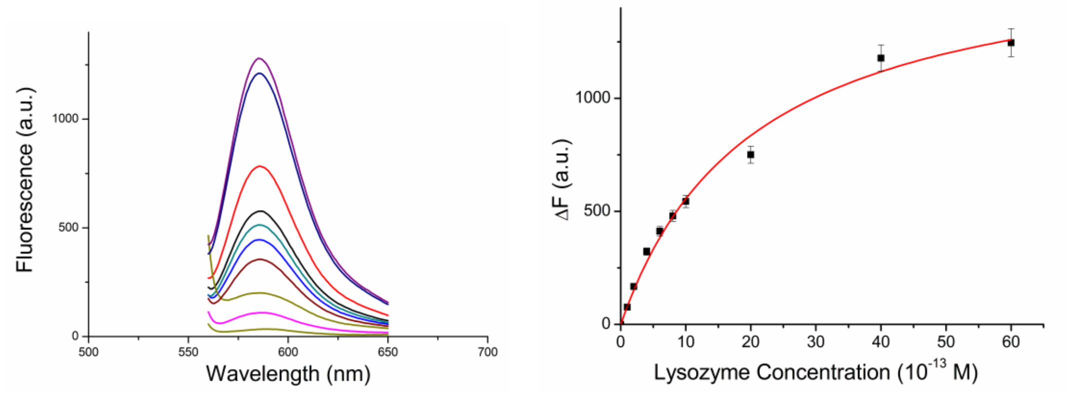

Figure S5 Fluorescence Curves of the whole molecular machine. From bottom to top: blank; 1.0×10^{-13} M; 2.0×10^{-13} M; 4.0×10^{-13} M; 6.0×10^{-13} M; 8.0×10^{-13} M; 1.0×10^{-12} M; 2.0×10^{-12} M; 4.0×10^{-12} M; 6.0×10^{-12} M;

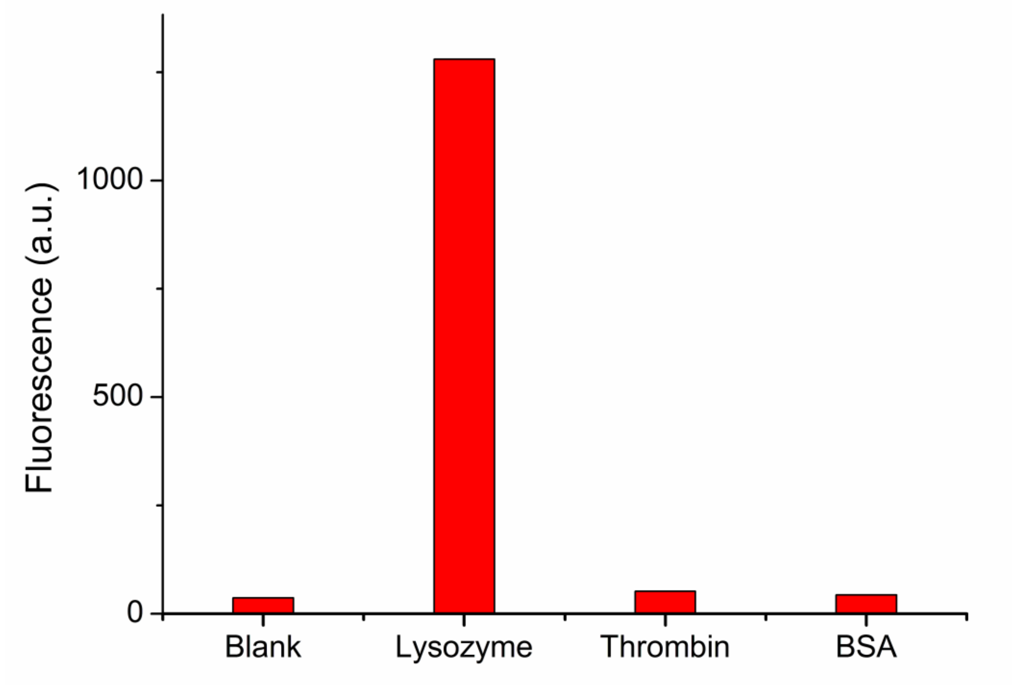

Figure S6. Selectivity of the molecular machine.

7. Selectivity

The molecular machine was initiated by the aptamer recognition of lysozyme, providing this protocol with selectivity. Experiments were carried out to investigate whether there was cross interference. To demonstrate this, lysozyme of low concentration (5.0×10^{-12} M) and two control analytes (thrombin and bovine serum albumin, BSA) of high concentration (5.0×10^{-10} M) were introduced into this molecular machine using the entire molecular machine. All the samples were tested according to the procedures depicted in sectrions S1.6 and S1.7. The results (Figure S6) showed that lysozyme yielded an obvious fluorescent readout, while readouts of thrombin and bovine serum albumin (BSA) were barely distinguishable from the blank response. A good selectivity was thus verified.

References in Supplementary Materials

[S1] Lizardi P M, Huang X, Zhu Z, Bray-Ward P, Thomas D C, Ward D C. Mutation detection and single-molecule counting using isothermal rolling-circle amplification[J]. *Nature Genetics*, 1998, 19: 225-232.
